# Sex Differences in the Association Between Dietary Pattern and Type 2 Diabetes Mellitus: Protocol for a Systematic Review

**DOI:** 10.2196/98958

**Published:** 2026-07-13

**Authors:** Marina Idalia Rojo-López, Ainoa López-Cortés, Maria Antentas, Georgina Pujolar-Díaz, Filip Bellon, Minerva Granado-Casas, Didac Mauricio

**Affiliations:** 1 Department of Endocrinology & Nutrition Institut de Recerca Sant Pau Barcelona, Catalonia Spain; 2 Centro de Investigación Biomédica en Red Diabetes y Enfermedades Metabólicas Asociadas Barcelona, Catalonia Spain; 3 Faculty of Medicine Universitat de Vic - Universitat Central de Catalunya Vic, Catalonia Spain; 4 Doctoral School Universitat de Vic - Universitat Central de Catalunya Vic, Catalonia Spain; 5 DAP-Cat Group, Unitat de Suport a la Recerca Barcelona Institut Universitari d'Investigació en Atenció Primària Jordi Gol Barcelona, Catalonia Spain; 6 Department of Nursing and Physiotherapy Universitat de Lleida Lleida, Catalonia Spain; 7 Research Group of Health Care (GreCS) Instituto de Investigación Biomédica de Lleida Lleida, Catalonia Spain

**Keywords:** type 2 diabetes mellitus, dietary pattern, diet, sex, gender, glucose

## Abstract

**Background:**

Diabetes mellitus encompasses disorders characterized by hyperglycemia due to pancreatic β-cell dysfunction. Type 2 diabetes (T2D) constitutes over 90% of cases, with a background of genetic, metabolic, and environmental risk factors. Knowing that sex differences impact insulin resistance and glycemic control, this review aims to identify differences in adherence to dietary patterns between women and men with T2D.

**Objective:**

This systematic review aims to evaluate sex differences in dietary pattern adherence among individuals with T2D and the implications of these differences for glycemic control.

**Methods:**

The protocol was developed using the PRISMA (Preferred Reporting Items for Systematic Reviews and Meta-Analyses) guidelines. Studies published until July 2026 will be identified by searching the following electronic databases: MEDLINE, Scopus, Cochrane Library, and Web of Science. Three investigators will independently screen articles based on titles and abstracts followed by a thorough analysis of selected full-text articles of interest. Articles on T2D and dietary pattern scores that include biological sex data will be included. The estimation of risk of bias will be performed using the Standard Quality Assessment Criteria for Evaluating Primary Research Papers From a Variety of Fields. To synthesize the results, a narrative analysis will be performed based on the GRADE (Grading of Recommendations Assessment, Development, and Evaluation) framework.

**Results:**

The search strategy was developed and refined between July 2025 and July 2026 through scoping and pilot searches. The comprehensive database search will be conducted in August 2026, covering records from database inception to July 2026. Study selection and data extraction are expected to be completed by December 2026, with publication anticipated in late 2027.

**Conclusions:**

This systematic review will provide a comprehensive overview of the role of sex in dietary adherence among individuals with T2D. Identifying sex-specific and gender differences may inform the development of tailored nutritional strategies and interventions aimed at improving glycemic outcomes. Ultimately, this work highlights the importance of incorporating sex-based approaches in the management of T2D to optimize long-term health outcomes.

**Trial Registration:**

PROSPERO CRD42024340213; https://www.crd.york.ac.uk/PROSPERO/view/CRD42024340213

**International Registered Report Identifier (IRRID):**

DERR1-10.2196/98958

## Introduction

Diabetes mellitus (DM) encompasses a heterogeneous group of disorders that have hyperglycemia in common [1]. Hyperglycemia results from different pathogenetic defects, although dysfunction or destruction of pancreatic β cells is a common feature of all forms of DM [2]. Type 2 diabetes (T2D) is the predominant type of DM, making up more than 90% of all DM cases [3]. The development of T2D is a complex process involving the interplay of genetic, metabolic, and environmental factors, resulting in β-cell dysfunction, insulin resistance, and subsequently elevated levels of blood glucose [4].

Sex is suggested to play a pivotal role in the development of insulin resistance and the onset of DM given that there is a higher prevalence of T2D among men, especially when compared to women before menopause [5]. Notably, even the DM risk assessment tool developed by the American Diabetes Association includes male sex among risk factors for DM [1]. The predisposition and clinical manifestation of DM differ between men and women due to differences in biological, cultural, lifestyle, environmental, and socioeconomic factors [6]. Although biological sex–related factors such as hormonal differences and adiposity distribution play a key role, gender-related determinants, including social roles, caregiving responsibilities, meal preparation practices, and socioeconomic inequalities, also significantly influence these differences, particularly in dietary behaviors [7-10]. At a biological level, this increased risk is thought to be related to differential patterns of adiposity storage [11]. It has been shown that men display superior self-care behaviors concerning physical activity and self-care maintenance, whereas women show more appropriate self-care practices for monitoring signs and symptoms but they experience poorer glycemic control [12]. However, in terms of dietary patterns and gender roles, women appear to take more of an active role in managing diet, whether for managing their own DM or caring for a family member with the disease [13]. In previous findings from our research group, we identified an association between higher Alternative Healthy Eating Index (AHEI) scores and female sex in individuals with T2D [14]. Furthermore, the influence of the Western dietary pattern is particularly pronounced in men, indicating an elevated risk of mortality associated with T2D [15].

To the best of our knowledge, there is a lack of a comprehensive review on sex-specific differences in the dietary patterns of individuals with T2D. This systematic review aims to provide a thorough synthesis of existing evidence, examining the nuanced interplay among sex, dietary patterns, and glycemic control in individuals living with T2D. By exploring this issue, we aim to contribute valuable insights that might inform future tailored interventions and strategies for both male and female people with T2D.

## Methods

This protocol was developed in accordance with the PRISMA-P (Preferred Reporting Items for Systematic Review and Meta-Analyses Protocols) 2015 statement [16].

### Study Aims

The aim of this systematic review is to evaluate sex differences in adherence to dietary patterns among individuals with T2D and assess whether these differences are associated with variations in glycemic control.

### Research Question

The research question was defined based on the population, intervention, comparator, outcome, and study design (PICOS) structured method: are there sex-based differences in dietary pattern adherence among individuals with T2D, and what are their implications for glycemic control?

The PICOS breakdown was as follows:

- Population: individuals with T2D, with sex-disaggregated data
- Intervention: adoption of dietary patterns
- Comparator: comparison between male and female individuals
- Outcome: adherence to dietary pattern scores (sex differences; primary outcome) and glycemic control indicators (hemoglobin A_1c_ [HbA_1c_] and fasting glucose; secondary outcomes)
- Study design: observational studies and secondary analyses of randomized controlled trial studies when relevant observational associations can be extracted

### Eligibility Criteria

#### Types of Studies

Studies will be selected for inclusion based on their relevance to addressing the PICOS domains. In the review, we will include cross-sectional, case-control, and prospective and retrospective cohort studies, as well as secondary analyses of randomized controlled trials or interventional studies if they (1) evaluate associations between sex and dietary patterns or intake and their impact on glycemic control in individuals with T2D, (2) describe sex as a variable or predictor of dietary patterns or intake that could have an impact on glycemic control, (3) were published in any language, and (4) appear in a peer-reviewed journal.

#### Population of Interest

Studies of women and men with T2D aged 18 years and older with a diagnosis of T2D will be included. Sex will be accepted as a biological characteristic of the participants caused by differences in sex chromosomes [6,17]. Studies that include individuals with other conditions will be included provided that the data for participants with T2D are reported separately. Moreover, as the terminology for describing T2D evolves, we will exclude articles that do not explicitly specify the type of DM. Only studies clearly stating that the participants meet the diagnostic criteria for T2D and adhere to the criteria current at the time of the study will be included.

The study population will include all patients, with no restrictions based on country, race, ethnic origin, toxic habits, antidiabetic medication or insulin use, and physical activity levels.

#### Primary Outcome: Adherence to Dietary Patterns

Dietary pattern refers to the habitual distribution of the quantity and variety of foods and beverages in a diet [18]. Validated dietary pattern scores will be used as the primary measure of adherence because they provide standardized and comparable assessments across studies using different dietary assessment methods [19,20]. This review will focus on dietary pattern scores used as a tool to measure adherence. Therefore, dietary patterns must have been assessed using validated indexes such as the AHEI, Healthy Eating Index, alternate Mediterranean diet score, Mediterranean Diet Adherence Score, Dietary Approaches to Stop Hypertension index, and others. Studies using different instruments will be grouped according to the dietary pattern assessed, and quantitative synthesis will only be performed when the exposure definition and scoring approach are sufficiently comparable. When comparability is insufficient, the findings will be synthesized narratively.

#### Secondary Outcome: Glycemic Control

The secondary outcome will be management of blood glucose levels, encompassing both low (hypoglycemia) and high (hyperglycemia) levels as defined by the international consensus report on glycemic control [21,22]. The parameters to evaluate glycemic control include HbA_1c_ and/or fasting blood glucose.

#### Exclusion Criteria

Studies will be excluded from this review if they only assess the consumption of a specific food or nutrient, do not use a scored index, use instruments that are not validated, or include only one 24-hour dietary recall to estimate dietary intake. Additionally, studies involving type 1 DM and pregnant or breastfeeding participants will be excluded. Furthermore, studies that meet the following criteria will be excluded: studies performed using animal models, in vitro and in vivo studies, studies without a defined outcome, studies with insufficient data to establish conclusions, reviews and other nonoriginal papers, studies whose full text is not available, and studies that do not report data stratified by sex. In the event that a study does not meet any of these criteria except for the latter, that is, not reporting data stratified by sex, the authors will be requested to provide HbA_1c_ and/or fasting glucose data stratified by sex, as well as diet score stratified by sex to be considered for inclusion if these data are available.

### Search Strategy

The search strategy will combine controlled vocabulary and search terms to identify studies in the following databases (from their inception to the most recent date): MEDLINE, Scopus, Web of Science, and the Cochrane Library. Studies published until July 2026 will be considered for inclusion. The search strategies will be adapted to the requirements of each database. We will also track the reference lists of relevant publications to identify additional studies.

This search will be conducted using the following search items (including Medical Subject Headings [MeSH] terms): (1) type 2 diabetes mellitus, (2) diet quality indices and dietary quality scores and (3) glycemic control-related outcomes (see Multimedia Appendix 1 for further details).

### Study Selection and Data Collection

The results of the search will involve the construction of a database consolidating the outcomes, aimed at identifying and eliminating duplicates. This database will serve as a centralized repository of our findings. The Rayyan program (Rayyan Systems Inc) will be used in blind mode to screen the articles. Initially, the articles will be screened based on their titles and abstracts.

Four investigators will independently undertake the task of selecting references from the gathered data. This rigorous multi-investigator assessment ensures a comprehensive and unbiased approach. Any disparities arising from their independent evaluations will be thoroughly discussed and reconciled through consensus. In the event of unresolved discrepancies, the final decision will be made by the principal investigator of the study.

To ensure a thorough database search, the search strategy will be tailored for each database (PubMed, Web of Science, Cochrane Library, and Scopus), conducted independently by 4 researchers. The first phase of screening, based on titles and abstracts, will be carried out by all 4 researchers. In the second phase, full-text screening will be conducted through a triangulation process in pairs, where each researcher will review 50% of the articles and then discuss their assessments with their assigned partner. Articles that remain in the “maybe” category after this stage will be discussed collectively by all 4 researchers. This ensures that the scrutiny remains consistent and comprehensive, enhancing the reliability of our research outcomes.

Data extraction will be performed using a summary table created in Microsoft Excel, which will include different variables such as the first author’s surname, year of publication, journal, study design, country, sample size, age, sex, T2D duration, HbA_1c_ and fasting blood glucose levels, dietary assessment methods, instruments used, statistical methods, and adjustment for confounders by sex. Moreover, the main outcomes of the study will be considered (glycemic control of individuals living with T2D and the adoption of healthy dietary patterns or intake) to answer the question of interest. We will extract effect estimates from the included studies to obtain data on odds ratios, risk ratio or relative risk, and/or hazard ratios for dichotomous outcomes and mean differences and SDs (with 95% CIs) for continuous outcomes.

Studies will be grouped for each synthesis according to the type of dietary index used, the outcome assessed, and the availability of sex-stratified data. Studies will be considered eligible for a given synthesis when their exposure, outcome definition, and effect measure are sufficiently comparable. When studies are too heterogeneous for quantitative pooling, they will be summarized narratively.

### Handling of Incomplete Data and Sex-Stratified Information

If relevant data are missing, unclear, or not reported separately by sex, the corresponding study authors will be contacted to request the missing information at least once via email, allowing for a minimum of 4 weeks for a response before the data are considered unavailable. If sex-disaggregated data cannot be obtained, the following approach will be applied hierarchically: (1) studies reporting only combined (nonstratified) estimates will be included in the main synthesis but flagged, and a sensitivity analysis will be conducted excluding them to assess their impact; (2) studies with partially missing outcome data will be included in the narrative synthesis if quantitative pooling is not appropriate; and (3) studies with insufficient data for any form of synthesis will be excluded from quantitative analyses and described narratively, with the reason for exclusion documented in a supplementary table. The proportion of studies affected by missing or nonstratified sex data will be reported and discussed as a limitation of the review.

### Risk-of-Bias Assessment

To address the risk of bias, the methodological quality of the included studies will be evaluated and scored according to the Standard Quality Assessment Criteria for Evaluating Primary Research Papers From a Variety of Fields [23]. Each checklist includes a series of questions with the response options “yes,” “partial,” “no,” or “not applicable” that evaluate key aspects such as study design, methodology, data analysis, and results. Reviewers will systematically assess each criterion, with a “yes” response indicating that the study meets the quality standards, whereas a “no” or “partial” response will suggest potential biases or methodological concerns.

Risk-of-bias assessment will be conducted independently by 2 reviewers for each included study. Discrepancies between reviewers will first be addressed through direct discussion aimed at reaching consensus. If consensus cannot be reached after discussion, a third reviewer will be included in the discussion, and their decision will be final. Interrater agreement will be reported using the Cohen κ coefficient to provide transparency about the consistency of the assessment process.

Rather than providing a numerical score, the checklist offers a structured evaluation to identify strengths and weaknesses. Study quality will be included in the final synthesis in two ways: (1) as a sensitivity analysis in which studies rated as having a high risk of bias are excluded to assess their influence on pooled estimates and (2) as a dimension within the GRADE (Grading of Recommendations Assessment, Development, and Evaluation) framework, where the risk of bias directly informs the certainty of evidence for each outcome (see the Summary of Findings and Certainty of Evidence section). The risk of bias will be visually presented using a traffic light plot.

Data synthesis will be performed using a summary table created in Microsoft Excel, which will include data extraction (see the Study Selection and Data Collection section) and the risk-of-bias assessment. Stata (StataCorp) will be used to perform the analyses described.

### Summary of Findings and Certainty of Evidence

We will rate the quality of evidence for primary outcomes according to the GRADE guidance from high to very low according to limitations in the “standards” domains (risk of bias, imprecision, inconsistency, indirectness, and publication bias) [24,25].

### Meta-Analysis and Effect Model

Whenever possible, data will be synthesized using a meta-analysis. Studies will be considered sufficiently comparable for quantitative synthesis when they involve similar populations, comparable dietary exposure or index definitions, equivalent outcome measures, and compatible effect estimates. Clinical, methodological, and statistical heterogeneity will be assessed through visual inspection of forest plots and interpretation of the *Q* and *I*^2^ statistics before pooling.

If the included studies are sufficiently homogeneous, a fixed-effects model will be used. In the presence of meaningful heterogeneity, a random-effects model will be applied using either the Cochran-Mantel-Haenszel or inverse variance method depending on the type of outcome and data available. If the studies are not sufficiently comparable or if heterogeneity is too high to justify pooling, the findings will be summarized narratively.

If a meta-analysis is not feasible, we will perform a subgroup analysis grouping studies based on the type of dietary index used.

### Sensitivity Analysis and Publication Bias

Data analysis will include a sensitivity analysis of the results considering the study designs and their associated risk of bias. If feasible, estimates obtained from the included studies about the association between biological sex and the prespecified outcomes will be pooled. When available, adjusted measures of association will also be pooled. Additionally, publication bias will be analyzed using a funnel plot, the Egger regression test, and imputation methods.

## Results

The systematic review will explore whether sex-based differences in dietary pattern adherence and glycemic control are reported among individuals with T2D. Any differences identified will be interpreted in light of both biological sex–related factors and gender-related social determinants, including caregiving roles, meal preparation responsibilities, and socioeconomic constraints.

The search strategy was developed and refined between July 2025 and July 2026, including preliminary scoping searches and pilot database searches to optimize the final search strategy. The comprehensive database search is planned for August 2026 and will cover records from database inception through July 2026, as specified in the protocol. As of July 2026, the review remained in the preparatory phase, and the final comprehensive database search, formal study selection, and data extraction had not yet been completed. Title and abstract screening, full-text review, and data extraction are expected to be completed by December 2026. Results are expected to be submitted for publication in late 2027. Funding information is provided in the Funding section.

## Discussion

### Expected Findings

This systematic review aims to explore the current evidence on sex differences and the association between dietary adherence and glycemic control in individuals with T2D, addressing the limited research available on how sex influences these factors in the context of T2D.

It is known that sexual dimorphism exerts a substantial influence on the requirements for nutrients, metabolic pathways, and susceptibility to diseases, being mediated by variables such as sex hormones, adipose tissue distribution, and several additional factors [26,27]. These biological differences lead to distinct metabolic reactions to various dietary components and patterns, underscoring the need for sex-specific considerations in nutritional guidelines; nevertheless, a considerable number of studies fail to incorporate analyses according to sex [28]. Considering that T2D is a major public health burden [29], it is crucial to explore sex differences within the framework of personalized medicine to achieve more efficient dietary management in T2D. However, it is important to acknowledge that observed differences between sexes may not be solely explained by biological factors. Gender roles, socioeconomic conditions, and cultural norms are also likely to shape dietary behaviors and, consequently, glycemic outcomes [30,31].

There is previous evidence suggesting that self-management practices differ by gender, impacting DM outcomes [30,31]. For example, among Latinx migrants with T2D, women often experience a unique combination of challenges, such as poverty, financial stress, depression, and social isolation, leading to poorer health outcomes [32]. In contrast, men are more likely to engage in excessive alcohol consumption, underscoring the need for mental health support [32]. Comprehensive care models addressing both physical conditions (eg, depression, T2D, and overweight) and social determinants (gender inequity, poverty, and cultural norms) are crucial for DM management [32]. Significant sex and gender role differences in DM care measures and self-management activities require tailored interventions: for instance, women benefit from dyslipidemia screening and exercise, whereas men need support in diet and glucose monitoring [33,34]. Furthermore, gender-related social roles and responsibilities, including greater involvement in meal preparation and dietary management, may influence adherence to healthy dietary practices and contribute to differences between men and women in DM self-care behaviors [31,35]. Addressing these disparities is crucial for improving health management and outcomes [36].

To our knowledge, this will be the first systematic review to specifically examine sex differences and their association with adherence to healthy dietary patterns, assessed through validated dietary indexes and glycemic control in individuals with T2D. We will not only synthesize the available evidence but also systematically map the gaps in sex-disaggregated data as well as gender role reporting in the nutritional epidemiology of T2D. These contributions have direct implications for the design of more equitable, personalized dietary interventions and for informing future research that adequately accounts for both biological sex– and gender-related determinants of dietary behavior.

### Limitations

This protocol anticipates several limitations. First, heterogeneity among the included studies is expected, particularly in the definitions of dietary adherence, glycemic control, and the dietary patterns evaluated. Variations in study designs and measurement tools may make comparisons difficult and limit generalizability. Second, the lack of sex-specific data or inconsistent disaggregation by sex may restrict the ability to analyze differences between men and women. Additionally, uncontrolled confounding factors, such as socioeconomic status, physical activity, pharmacological treatments, and comorbidities, may influence outcomes but not be consistently reported. Furthermore, some treatments for T2D may require specific dietary modifications, supplementation, or meal distribution strategies, which could alter dietary patterns and glycemic control outcomes. Publication bias may lead to the omission of relevant evidence; however, no language restrictions will be applied in this review. Finally, significant heterogeneity may limit the feasibility of a meta-analysis, requiring reliance on narrative synthesis. Despite these limitations, the review aims to provide valuable insights into sex-specific differences in dietary adherence and glycemic control outcomes in individuals with T2D.

### Comparison With Prior Work

Previous studies have indicated that men and women with DM differ significantly in their adherence to dietary recommendations [37]. Women tend to follow healthier diets more consistently than men [38]. However, it has been reported that women tend to consume more sugar [6]. Moreover, a recent meta-analysis found that both sexes faced an increased risk of T2D with a high glucose index diet. However, women showed a higher risk than men after adjusting for factors such as carbohydrates, ethnicity, and follow-up duration [39]. Another cross-sectional study in Brazil and Venezuela observed worse glycemic control in women with T2D compared to men [40].

However, regarding dietary patterns, an observational study found that women with T2D consumed higher amounts of legumes, vegetables, fruits, and vegetable oils than men, showing a better dietary profile [37]. Another observational study found that Spanish women with T2D had the highest scores on the AHEI and Mediterranean Diet Adherence Score [14]. Similarly, in the PREDIMED clinical trial, women showed greater adherence to the Mediterranean diet pattern [41].

Furthermore, we need to highlight that women with DM often prioritize their family’s needs over their own, whereas men are more likely to depend on female relatives for dietary management [13]. Indeed, in a qualitative study, women reported receiving less social support for managing T2D, including dietary adherence [42]. Men are less likely to oversee diet in general [13]. On the one hand, women tend to eliminate unhealthy foods from their diet, although they sometimes lie about their eating habits. On the other hand, men prefer to moderate the consumption of certain foods within the family environment, even promoting changes in the rest of the household’s diet supported by their wives [13]. However, outside this context, men tend to not modify their habits, which leads them to limit activities that could jeopardize dietary recommendations. Female relatives are often more involved in managing a patient’s diet, whereas male relatives provide support but are less likely to monitor diet closely. Furthermore, men are usually more autonomous in managing their DM, adjusting their physical activity and diet to better align with their medication regimen, which may enhance self-management [42].

### Conclusions

In conclusion, this systematic review aims to address the critical role of sex differences in dietary adherence and glycemic control among individuals with T2D. Gender-responsive strategies enable health care professionals to provide personalized care, particularly regarding dietary patterns, to optimize T2D management. These approaches help identify the specific needs of men and women, improving long-term health outcomes. Furthermore, it will be important in the future to explore how hormonal, psychological, and social factors influence self-care behaviors and disease management.

## Data Availability

The datasets used and/or analyzed during this study are available from the corresponding author on reasonable request.

## Appendix

Multimedia Appendix 1 Search conducted in PubMed (adapted for each database).

## Abbreviations

AHEI: Alternative Healthy Eating Index
DM: diabetes mellitus
GRADE: Grading of Recommendations Assessment, Development, and Evaluation
HbA1c: hemoglobin A1c
MeSH: Medical Subject Headings
PICOS: population, intervention, comparator, outcome, and study design
PRISMA-P: Preferred Reporting Items for Systematic Review and Meta-Analyses Protocols
T2D: type 2 diabetes
