# Supplementary material for: Sex Differences in the Association Between Dietary Pattern and Type 2 Diabetes Mellitus: Protocol for a Systematic Review
Source: JMIR Res Protoc. 2026 Jul 13;15:e98958. doi: 10.2196/98958 (PMC13408464; doi:10.2196/98958)
Supplement: Multimedia Appendix 1 [file resprot_v15i1e98958_app1.docx]

## **Appendix 1**

**Search conducted in PubMed (adapted for each database):**

(

("Diabetes Mellitus, Type 2"[MeSH] OR "type 2 diabetes"[tiab]

OR "type 2 diabetes mellitus"[tiab] OR T2D[tiab] OR T2DM[tiab]

OR "non-insulin-dependent diabetes"[tiab] OR NIDDM[tiab])

AND

("Diet, Healthy"[MeSH] OR "Diet, Mediterranean"[MeSH]

OR "Diet, Plant-Based"[MeSH] OR "Feeding Behavior"[MeSH]

OR "diet quality"[tiab] OR "dietary quality"[tiab]

OR "diet score"[tiab] OR "dietary score"[tiab]

OR "dietary pattern*"[tiab] OR "eating pattern*"[tiab]

OR "food pattern*"[tiab] OR "diet pattern*"[tiab]

OR "dietary index"[tiab] OR "dietary indices"[tiab]

OR "diet index"[tiab] OR "diet indices"[tiab]

OR "healthy eating index"[tiab] OR "HEI"[tiab]

OR "alternative healthy eating index"[tiab] OR "AHEI"[tiab]

OR "Mediterranean diet"[tiab] OR "Mediterranean diet score"[tiab]

OR "alternate Mediterranean diet"[tiab] OR "aMED"[tiab]

OR "DASH diet"[tiab] OR "dietary approaches to stop hypertension"[tiab]

OR "DASH score"[tiab]

OR "dietary inflammatory index"[tiab] OR "DII"[tiab]

OR "empirical dietary inflammatory pattern"[tiab] OR "EDIP"[tiab]

OR "MIND diet"[tiab]

OR "plant-based diet*"[tiab] OR "plant-based dietary index"[tiab]

OR "PDI"[tiab] OR "healthful plant-based diet"[tiab] OR "hPDI"[tiab]

OR "prudent diet"[tiab] OR "western diet"[tiab]

OR "dietary diversity"[tiab] OR "dietary diversity score"[tiab]

OR "overall diet quality"[tiab] OR "diet adherence"[tiab]

OR "dietary adherence"[tiab])

AND

("Glycated Hemoglobin"[MeSH] OR "Blood Glucose"[MeSH]

OR "glycemic control"[tiab] OR "glycaemic control"[tiab]

OR "glycemic management"[tiab] OR "glycaemic management"[tiab]

OR "glycemic target*"[tiab] OR "glycaemic target*"[tiab]

OR HbA1c[tiab] OR "hemoglobin A1c"[tiab] OR "haemoglobin A1c"[tiab]

OR "glycated hemoglobin"[tiab] OR "glycosylated hemoglobin"[tiab]

OR "fasting glucose"[tiab] OR "fasting plasma glucose"[tiab]

OR "postprandial glucose"[tiab] OR "blood glucose"[tiab])

)

NOT

("pregnancy"[tiab] OR "pregnant"[tiab] OR "gestational diabetes"[tiab]

OR "lactation"[tiab] OR "gravidarum"[tiab]

OR "type 1 diabetes"[tiab] OR T1DM[tiab] OR T1D[tiab]

OR "Diabetes Mellitus, Type 1"[MeSH])
